# Nontraumatic unilateral ureteral entrapment at the sacroiliac joint: A rare cause of obstructive uropathy

**DOI:** 10.1016/j.radcr.2026.06.167

**Published:** 2026-07-24

**Authors:** Rushikesh Dave, Kanan Panchal, Naitik Patel, Dharita Shah

**Affiliations:** Department of Radiognosis, Sardar Vallabhbhai Patel Institute of Medical Science and Research, Smt. N.H.L. Municipal Medical College, Ahmedabad, India

**Keywords:** Ureteral entrapment, Sacroiliac joint, Obstructive uropathy, Computed tomography, Urography, Degenerative osteoarthritis

## Abstract

Ureteral entrapment within the sacroiliac joint is an exceedingly rare anatomical phenomenon. To date, 6 cases have been documented in the literature: 1 cadaveric and 5 clinical reports. Among the clinical cases, 3 followed high-energy pelvic trauma, while 2 were nontraumatic. We report the third nontraumatic clinical case (and seventh case overall) in a 77-year-old woman presenting with chronic lower back pain. Computed tomography intravenous pyelography revealed focal entrapment of the right ureter at the level of the sacroiliac joint due to advanced degenerative sacroiliitis. Despite a 10-12 hours delay in contrast excretion, the patient was urologically asymptomatic with stable renal function. Unlike previous nontraumatic cases requiring surgical intervention or nephrostomy, this patient was managed conservatively.

## Introduction

Ureteral entrapment within the sacroiliac joint (SIJ) remains a clinical rarity, with only 6 cases documented in the literature [[1], [2], [3], [4], [5]]. Historically, the majority of these instances followed high-energy pelvic ring disruptions [[1], [2], [3],^6^], making nontraumatic cases in the geriatric population exceptionally rare [4,5].

We report a case of unilateral, nontraumatic ureteral entrapment in a 77-year-old female. While previously reported nontraumatic cases necessitated surgical or percutaneous intervention, our patient was managed conservatively due to stable renal function and an absence of urological symptoms. Herein, we describe the imaging features of this rare entity and review the existing literature.

## Case report

A 77-year-old female presented with a long-standing history of lower back pain. She denied any primary urinary complaints, including hematuria, dysuria, or changes in voiding frequency. Physical examination revealed tenderness over the SIJ but was otherwise unremarkable.

**Fig. 1:**
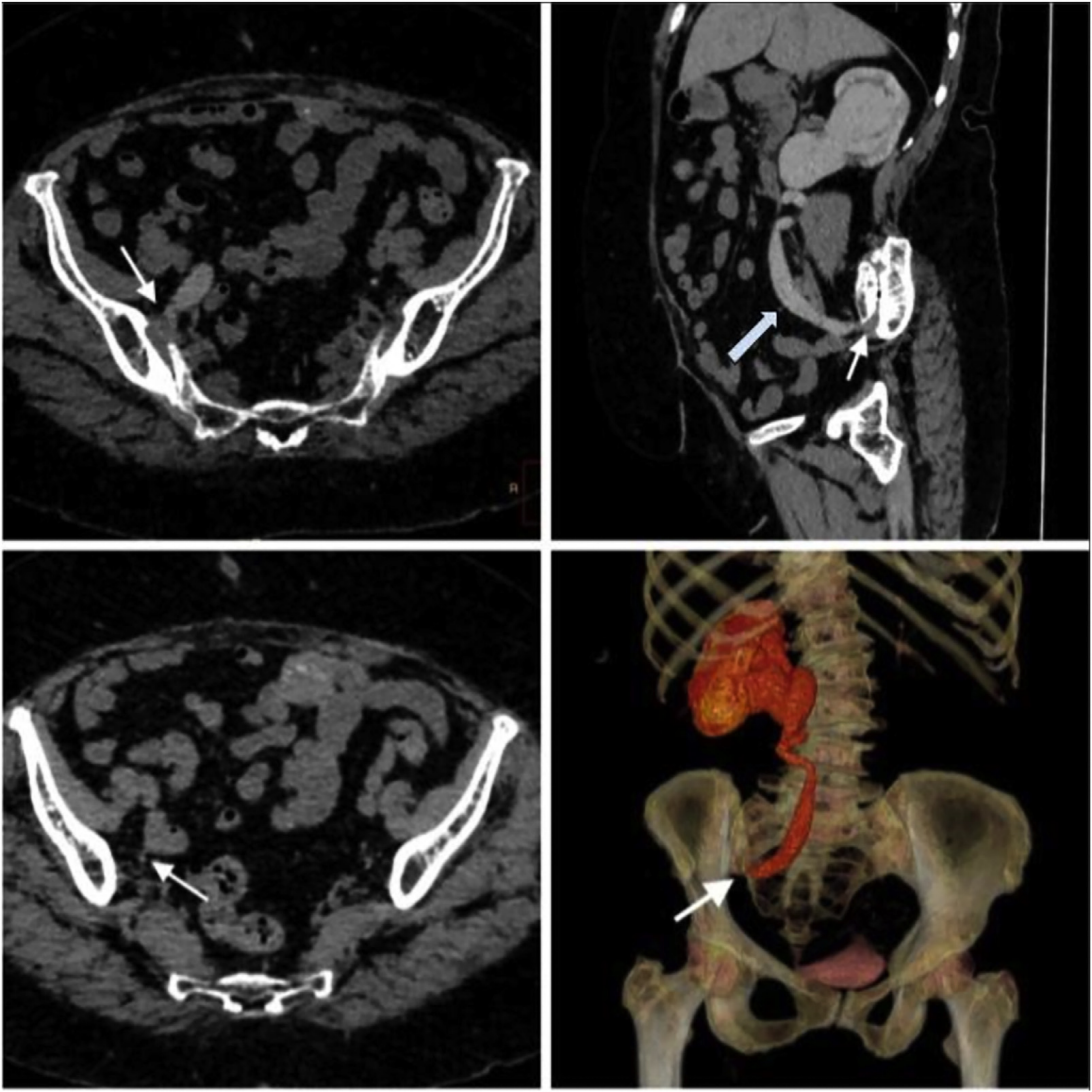
Nontraumatic right ureteral entrapment at the sacroiliac joint in a 77-year-old female. (A) Axial contrast-enhanced CT intravenous pyelography (CT IVP) image at the level of the sacroiliac joints. There is moderate dilatation of the right ureter (white arrow) terminating abruptly at the anterior aspect of the right sacroiliac joint. (B) Sagittal reformatted CT image clearly visualizing the focal narrowing and entrapment of the right ureter within the irregular margins of the degenerative sacroiliac joint (white arrow) and dilated proximal ureter (long arrow) obtained on 12 hours scan. (C) Axial CT image through the lower pelvis, distal to the site of entrapment. The distal right ureter appears narrow and nondilated (white arrow), confirming the level of obstruction. (D) Three-dimensional (3D) volume-rendered CT reconstruction of the image obtained after 12 hours was done using Philips intellispace portal providing a global view. The dilated right ureter is seen descending and terminating at the level of the right sacroiliac joint (white arrow). Note the associated ureteral kinking superiorly.

Initial screening with ultrasonography (USG) demonstrated mild right-sided hydronephrosis with a dilated upper ureter. The serum creatinine of the patient was 1.5 mg/L. The urine routine microscopy did not reveal any significant abnormality. To further investigate the etiology, a contrast-enhanced computed tomography (CT) intravenous pyelography was performed on a 128-slice Philips CT scanner machine using 5 mm thick and 1 mm thin contiguous axial scan of kidneys, ureter and bladder with I.V. contrast using single bolus 3—phase protocol. In the first phase, a noncontrast plain scan of the abdomen and pelvis was taken. 60 mL (1 mL/kg) nonionic contrast was injected at a rate of 3 mL/s^2^ after a negative test dose. The Nephrogram and excretory phase were then obtained after approximately 80 seconds and 10 minutes of contrast injection respectively. Consecutive prolonged delayed phases were taken at 30 minutes, 60 minutes, 3 hours, and 10-12 hours. The imaging confirmed mild-to-moderate right hydronephrosis with mild cortical thinning and mild-to-moderate hydroureter, with the ureter appearing dilated upto the level of right sacroiliac joint. At this level, the ureter was adherent to the SIJ, with a sudden change in caliber and narrowing noted immediately distal to the joint (Fig. 1). There was no evidence of an intraluminal calculus, an enhancing mass lesion, extrinsic compression, or surrounding fibrosis to account for the sudden narrowing.

**Fig. 2:**
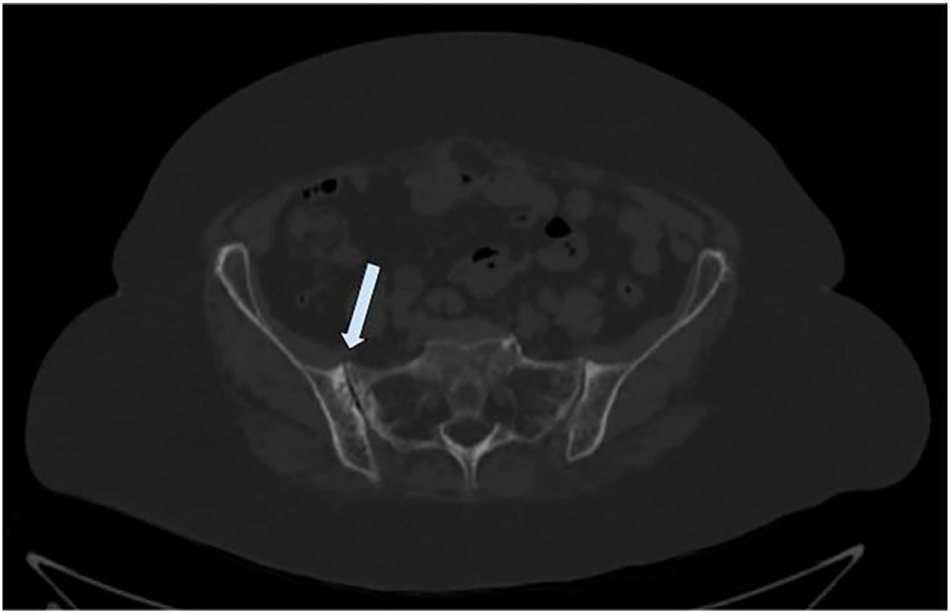
Degenerative changes in the right sacroiliac joint. Axial CT image of bone window through the pelvis shows degenerative changes in the form of lytic and sclerotic changes in the right sacroiliac joint (white arrow).

Significant secondary findings included a Grade III (severe and retrograde) kinking of the proximal ureter at the L3-L4 vertebral level, categorized using the CT urography classification system described by Kamo et al [7]. Consistent with the literature, this finding was found to be a benign physiological consequence of ureteral mobility within the perirenal space at the gonadal vein crossing point. The kinking of the ureter did not cause mechanical obstruction or proximal hydroureteronephrosis. Instead, these findings resulted from a downstream caliber change at the right sacroiliac joint. The SIJ exhibited advanced degenerative changes, including bilateral lytic–sclerotic margins and reduced joint space, more pronounced on the right side (Fig. 2). Functionally, the right kidney showed delayed, persistent enhancement with contrast excretion markedly delayed until 10-12 hours postinjection, confirming a significant obstructive uropathy. The imaging findings were consistent with nontraumatic ureteral entrapment at the right SIJ secondary to chronic degenerative sacroiliitis.

## Discussion

The ureter’s descent anterior to the psoas muscle and its transit across the pelvic brim directly over the SIJ represents a critical anatomical “bottleneck.” At this junction, the ureter is relatively fixed, making it vulnerable to extrinsic compression if the underlying joint architecture is altered. While radiology literature typically focuses on intraluminal or neoplastic causes of obstruction, the proximity of the ureter to the bony pelvis remains a significant, albeit overlooked, factor in complex cases.

Historically, ureteral entrapment within the SIJ has been documented as a sequela of high-energy pelvic ring disruptions, where the ureter becomes incarcerated within a fracture diastasis or an anterior joint dislocation [2,8]. More recently, orthopedic reports have highlighted similar risks in specific acetabular fractures, where the ureter may become trapped between displaced bone fragments [9].

However, nontraumatic entrapment—as seen in our 77-year-old patient—is exceedingly rare. Only 2 such clinical cases have been reported: Yeung et al. [4] described an “idiopathic” bilateral entrapment in an elderly multiparous patient, and Lee and Cho [5] reported a unilateral case that mimicked the appearance of tuberculous ureteritis. In both prior cases, the clinical presentation necessitated intervention—Yeung et al. [4] utilized percutaneous nephrostomy for renal impairment, while Lee and Cho [5] performed a surgical ureteral excision (Table 1).

**Table 1:** Summary of previously reported cases of ureteral entrapment within the sacroiliac joint.

Study	Type	Cause	Side	Imaging modality	Treatment	Outcome
Noakes et al. [1]	Traumatic	Blunt pelvic trauma (realignment of SIJ)	Left	IVP, radioisotope renal scan, USG, and RGP/AGP	Ureteroneocystostomy with ureteral trimming and a psoas hitch	Prompt renal function with residual Cali ectasis
Konety et al. [2]	Traumatic	Blunt abdominal trauma (pubic and SIJ fracture)	Unilateral	N/A (intraop)	Ureter released from fractures	No ureteral damage was observed
Shetty et al. [3]	Traumatic	Pelvic trauma (pelvic fracture)	Unilateral	N/A (postlaparotomy)	Details not provided	Details not provided
Ahmed et al. [9]	Traumatic	Trauma (acetabular fracture)	Left	Radiographs CE-CT with 3D reconstructions	Ureter removal from fracture and ureteral stenting	Asymptomatic on 7 mo postop
Yeung et al. [4]	Nontraumatic	Idiopathic; (joint laxity and osteophytes)	Bilateral	Cystoscopy, AGP, and CT	Managed conservatively with PCN as surgery was declined	Normal follow-up creatinine levels
Otsuru et al. [6]	Nontraumatic	Idiopathic; (cadaver study)	Left	N/A (dissection)	N/A (cadaveric study)	Left renal hypertrophy and hydroureter
Lee and Cho [5]	Nontraumatic	Idiopathic; (degenerative)	Right	USG, IVP, RGP, and dynamic CT	Ureter excision and end-to-end anastomosis	The patient recovered well

AGP, antegrade pyelograms; CECT, contrast enhanced computed tomography; CT, computed tomography; IVP, intravenous pyelography; N/A, not applied; PCN, percutaneous nephrostomy; RGP, retrograde pyelograms; SIJ, sacroiliac joint; USG, ultrasonography.

In contrast, our patient was treated conservatively in view of advanced age, the chronic nature of the obstruction, and the relative stability of her renal function. The patient was informed of the findings and advised to undergo a DTPA scan which she refused and was kept under close urological surveillance with periodic renal ultrasonography to monitor for any progression of hydronephrosis. After the diagnosis, one follow up ultrasonography study after 3 months was done which showed no significant progression in hydronephrosis. The patient was advised to undergo further periodic checkups in the future for monitoring.

This case serves as a vital reminder to maintain a high index of suspicion for non-traumatic ureteral entrapment within the SIJ when evaluating hydronephrosis of unknown etiology. While rare, this mechanical obstruction can be identified by tracing the ureter’s path as it traverses the degenerative joint space. Early recognition of this entity is essential to ensure timely, appropriate management and to avoid unnecessary invasive procedures in the elderly population.

## Authors’ contribution statement

Rushikesh Dave was responsible for case conception, primary imaging interpretation, and drafting of the manuscript. Naitik Patel assisted in drafting and editing manuscript. Kanan Panchal assisted in critical revision of the manuscript for intellectual content. The study was done under guidance of Dharita Shah who assisted in the intellectual content of the article. All authors have read and approved the final version.

## Patient consent

The authors declare that informed and written consent was obtained from the patient for the publication of this case report and any accompanying radiological images and clinical data.
